# Association Between Adipose Tissue Proton Density Fat Fraction, Resting Metabolic Rate and FTO Genotype in Humans

**DOI:** 10.3389/fendo.2022.804874

**Published:** 2022-02-28

**Authors:** Theresa Drabsch, Daniela Junker, Sandra Bayer, Mingming Wu, Cora Held, Dimitrios C. Karampinos, Hans Hauner, Christina Holzapfel

**Affiliations:** ^1^ Institute for Nutritional Medicine, School of Medicine, Technical University of Munich, Munich, Germany; ^2^ Department of Diagnostic and Interventional Radiology, School of Medicine, Technical University of Munich, Munich, Germany; ^3^ Else-Kroener-Fresenius Centre of Nutritional Medicine, Chair of Nutritional Medicine, School of Life Sciences, Technical University of Munich, Freising-Weihenstephan, Germany

**Keywords:** energy expenditure, genotype, *FTO*, brown adipose tissue, proton density fat fraction

## Abstract

**Background:**

The difference of proton density fat fraction (PDFF) between supraclavicular and gluteal adipose tissue might indicate the presence of brown adipose tissue (BAT). Aim of this cross-sectional study was to investigate the association between PDFF over the supraclavicular fat region as a proxy of BAT proportion and resting metabolic rate (RMR). In addition, the association between the single nucleotide polymorphism (SNP) rs1421085 at the *fat mass and obesity associated* (*FTO*) gene locus and both PDFF and RMR was investigated.

**Methods:**

Anthropometric, clinical, and lifestyle data from 92 healthy adults (66.3% females, mean age: 36.2 ± 13.0 years, mean body mass index: 24.9 ± 5.4 kg/m^2^) were included in the analysis. The RMR was measured by indirect calorimetry. The magnetic resonance imaging (MRI) was used for the measurement of visceral and subcutaneous adipose tissue (VAT, SAT) volumes and for the measurement of adipose tissue PDFF.

**Results:**

Mean RMR of the whole group was 1 474.8 ± 242.2 kcal. Genotype data was available for 90 participants. After adjustment for age, sex, weight change and fat-free mass (FFM), no association was found between supraclavicular PDFF (p = 0.346) and gluteal PDFF (p = 0.252), respectively, and RMR, whereas statistically significant evidence for a negative association between delta PDFF (difference between gluteal PDFF and supraclavicular PDFF) and RMR (p = 0.027) was obtained. No statistically significant evidence was observed for per *FTO* risk allele change in RMR, gluteal and supraclavicular PDFF maps or volumes of VAT and SAT.

**Conclusions:**

Supraclavicular PDFF as a surrogate marker of BAT presence is not a determinant of RMR under basal conditions. In the present study, the *FTO* rs1421085 variant is not associated with either RMR or PDFF. Further studies are needed to elucidate the effect of BAT on RMR.

## Introduction

Understanding the determinants that influence resting metabolic rate (RMR) as the main component of energy metabolism is fundamental in obesity research. Main determinants of RMR variability include fat-free mass (FFM), sex, age, and body temperature (1–3). In particular, FFM including muscle mass and solid organs explain up to 80% of RMR variability (1, 3–6). However, also fat mass (FM), depending on its extent and location, may play a role in RMR variability, although FM shows a 6 to 7 times lower specific metabolic rate than FFM (7).

The FM consists of white adipose tissue (WAT), composed of lipid-storing white and – to a poorly defined, but much lower extent - lipid-burning “beige” adipocytes, and brown adipose tissue (BAT) in specific depots (8, 9). Whereas WAT stores energy as triglycerides, BAT and the beige fat cells within WAT produce heat by the expression of uncoupling protein 1 (UCP1) (8).

Over the last decade, functional imaging provided evidence for the existence of BAT in adult humans (8), where BAT is mostly found in the supraclavicular and neck region (8, 10, 11). In most studies, positron-emission tomography and computed tomography (PET-CT) was used to identify active BAT (10). More recently, water-fat magnetic resonance imaging (MRI) was applied to detect BAT (12–14). One possibility for the surrogate detection of the presence of brown and beige adipose tissue by MRI is the quantification of the proton density fat fraction (PDFF), which is defined as the proportion of mobile proton density from triglycerides over the total density of mobile protons from triglycerides and mobile water (15). In a recent study, supraclavicular PDFF was positively associated with anthropometric obesity markers as well as volumes of visceral and subcutaneous adipose tissue (VAT, SAT) (16). Calculating the difference between the mean supraclavicular PDFF and mean gluteal PDFF values (delta PDFF) has been proposed with the aim of detecting BAT presence. Referencing the supraclavicular PDFF to the gluteal PDFF would partially alleviate the impact of obesity on a generally lower adipose tissue hydration. It has been shown that the higher the delta PDFF was, the more likely BAT was detected (14, 17, 18).

Although still controversially debated, the presence of active BAT may play a significant role in human energy metabolism. Studies have shown that at least under stimulation, such as cold exposure, BAT is activated and RMR is transiently increased (19–21). Recently, Claussnitzer et al. identified a causal relationship between the single nucleotide polymorphism (SNP) rs1421085 at the *fat mass and obesity associated* (*FTO*) locus and preadipocyte differentiation (22). Thereby, the risk variant may lead to an enhanced fat storage, due to a cell-autonomous change of adipogenesis towards white adipocytes.

The aim of the current analysis was to investigate (I) the association between supraclavicular PDFF, as a surrogate marker for the presence of BAT, and RMR as well as (II) associations between the *FTO* SNP rs1421085 and PDFF including delta PDFF and RMR.

## Materials and Methods

### Subjects

Recruitment of study participants started in October 2013 at the Else Kroener-Fresenius Centre for Nutritional Medicine in Munich, Technical University of Munich, Germany. Adults with a body mass index (BMI) equal to or above 18.5 kg/m² were included. Participants were excluded if an acute inflammatory state, a history of severe diseases, surgeries within the last three months or physical limitations were reported. Pregnant and breast-feeding women and persons on specific medications such as beta-blocker were also excluded. Written informed consent was obtained before participation in the study. The study protocol and the standard operating procedures were approved by the ethical committee of the Technical University of Munich, Germany (Number 2719/10 S).

### Anthropometry

Height was measured in a standing position without shoes using a stadiometer (Seca, Hamburg, Germany) and reported to the nearest 0.1 cm. Body composition, including FFM and FM, was assessed using bioimpedance analysis (BIA; Tanita BC 418 MA, Tokyo, Japan). Measurements were performed before indirect calorimetry in light clothing and with voided bladder, with subtraction of 1.0 kg for clothes. The BMI was calculated as quotient of weight in kg and squared height in m (kg/m²). Blood pressure and heart rate were obtained using a validated electronic device (Beurer BM70, Ulm, Germany) according to the recommendations of the American Heart Association (23).

### RMR Measurement

Indirect calorimetry was applied for the measurement of RMR with a ventilated canopy hood (Quark RMR, Suite Version 10.0e, Cosmed s.r.l., Rome, Italy). The RMR measurement was performed with the following requirements: overnight fast (10 to 12 hours), no smoking, and abstinence from physical activity the day before. The 30-minutes measurement started after an acclimatization time of five minutes. The flow rates were assessed as recommended by the manufacturer. The Quark RMR software analyzed oxygen consumption (VO_2_) and carbon dioxide (VCO_2_) expiration resulting from substrate oxidation, while participants were fully awake and motionless. The respiratory quotient (RQ) was calculated as VCO_2_/VO_2_ ratio. For RMR calculation, the following equation was used: RMR (kcal/d) = [3.941 × VO_2_ (ml/min) + 1.106 × VCO_2_ (ml/min)] × 1.44 (24). The VO_2_ and/or VCO_2_ variances equal to or greater 10.0% were re-calculated by cutting out a four-minute window for correction (25). The results using a 4-minutes time window were not statistically different to that measured over a 30-minutes period (25). If correction was not feasible, participants were excluded from the analysis.

### MRT Measurement

Participants were invited for MRI scanning. Exclusion criteria included standard contraindications for MRI examinations. All participants had given written informed consent before the examination and underwent an MRI of the neck and the abdomen/pelvis on a 3T Philips scanner (Ingenia, Philips Healthcare, Best, The Netherlands). For PDFF analysis, a semi-automatic segmentation tool based on Matlab (MathWorks, Natick, MA, USA) was used. Weight data at the time of MRI measurement was self-reported. Detailed data analysis of MRI measurement was performed as previously described (16). The processing steps were followed to determine SAT volume, VAT volume, mean supraclavicular and gluteal PDFF.

### Genotyping

Deoxyribonucleic acid (DNA) was isolated from EDTA full blood samples according to a standard protocol following manufacturer`s protocol (DNeasy Blood & Tissue Kit, QIAGEN, Hilden, Germany). Polymerase chain reaction (PCR) was used for amplification of DNA. Sequencing of the *FTO* polymorphism rs1421085 was carried out by the company Eurofins (Eurofins Genomics GmbH, Ebersberg, Germany) with Cycle Sequencing Technology (dideoxy chain termination/cycle sequencing) using Applied Biosystems 3730xl DNA Analyser. Call rate was calculated with 96.1% showing a high quality of the genotyping procedure. Calculated minor allele frequency (MAF) of the risk C allele was 43%. Hardy-Weinberg equilibrium (HWE) was fulfilled.

### Data Analysis

Results of the descriptive analysis were expressed as means ± standard deviations (SDs) and percentages (%). For comparison of variables, the unpaired student t-test was conducted for normally distributed variables, otherwise Mann-Whitney U-test was used. Analysis of variance (ANOVA) were conducted to compare more than two variables. Regression analysis was calculated for associations between different variables. Additive, as well as dominant and recessive linear models were used to identify associations between the *FTO* genotype and PDFF and RMR. Due to time intervals between the RMR and MRI measurement, all statistical analysis were adjusted for the body weight difference (“weight change”) between the time of RMR and MRI measurement. RMR is known to be significantly associated with age, sex, and FFM (1, 3, 26, 27). For these reasons, three different models were applied: model 1 was unadjusted, model 2 was adjusted for age, sex, FFM, and weight change, model 3 contained model 2 and further adjustment for FM. Due to the explorative character of the study, no correction for multiple testing was performed. All estimates were presented as unstandardized betas with 95% confidence intervals (CI). P-values lower than 0.05 were considered as statistically significant. All statistical analyses were performed by using the statistical software R (R Core Team, 2019) (28).

## Results

### Baseline Characteristics

In total, 92 participants (66.3% female) were included in the analysis (**Table 1**). Mean age was 36.2 ± 13.0 years and mean BMI was 24.9 ± 5.4 kg/m² (range, 18.5 - 43.1 kg/m²). Mean RMR was 1 474.8 ± 242.2 kcal/d, with males (1 711.0 ± 213.0 kcal/d) having on average a 355 kcal/d higher RMR than females (1 356.0 ± 152.7 kcal/d) (p ≤ 0.001). Mean supraclavicular and gluteal PDFF were 76.0 ± 4.7% and 89.3 ± 3.9%, respectively. Supraclavicular PDFF was significantly lower than gluteal PDFF (p ≤ 0.001) with a mean delta PDFF (difference between gluteal PDFF and supraclavicular PDFF) of 13.3 ± 4.2%. No sex-specific differences between PDFFs, delta PDFF, VAT, or SAT were detected. Almost 50% (48.9%) of the participants (n = 44) were heterozygous for the C risk allele of the FTO SNP rs1421085, while 20.0% of the participants (n = 18) were homozygous for the C risk allele (**Table 1**).

**Table 1:** Characteristics of the study population.

Parameter	n (%)	Mean ± SD	Min	Max
Age (years)	92	36.15 ± 13.04	20.00	69.00
Weight (kg)	92	73.87 ± 16.62	50.30	134.30
Height (cm)	92	172.30 ± 9.70	155.20	195.00
BMI (kg/m²)	92	24.89 ± 5.37	18.48	43.08
*BMI categories*				
18.5-24.9	56 (60.87)			
25.0-29.9	25 (27.17)			
≥30	11 (11.96)			
PDFF sup. (%)	92	76.01 ± 4.71	66.51	87.17
PDFF glut. (%)	92	89.27 ± 3.86	73.44	97.28
Delta PDFF (%)	92	13.26 ± 4.24	2.56	22.70
VAT (ml)	89	1 258.08 ± 1 353.06	81.63	6 511.86
SAT (ml)	89	6 114.30 ± 4 349.86	758.50	22 425.90
FM (%)	92	25.66 ± 10.88	4.10	51.40
FM (kg)	92	19.63 ± 11.50	3.20	56.00
FFM (kg)	92	54.26 ± 11.91	37.60	86.90
RMR (kcal/d)	92	1 474.8 ± 242.2	1 022.00	2 123.00
VO_2_ (ml/min)	92	213.47 ± 35.85	149.00	309.00
VCO_2_ (ml/min)	92	175.89 ± 28.67	116.00	251.00
RQ	92	0.82 ± 0.06	0.68	0.96
Systolic BP (mmHg)	90	116.93 ± 15.54	95.00	199.00
Diastolic BP (mmHg)	90	71.61 ± 8.69	52.00	96.00
Heart rate (bpm)	90	60.39 ± 9.15	41.00	89.00
*FTO rs1421085* (n=90)				
Homozygous CC	18 (20.00)			
Heterozygous CT	44 (48.89)			
Homozygous TT	28 (31.11)			

Mean ± SD is shown, min and max are given, if not otherwise indicated; BMI, body mass index; BP, blood pressure; bpm, beats per minute; delta PDFF, difference between PDFF glut. and PDFF sup.; FFM, fat-free mass; FM, fat mass; FTO, fat mass and obesity associated; glut., gluteal; n, number; PDFF, proton density fat fraction; RMR, resting metabolic rate; RQ, respiratory quotient; SAT, subcutaneous adipose tissue; SD, standard deviation; sup., supraclavicular; VAT, visceral adipose tissue; VCO_2_, volume of carbon dioxide; VO_2_, volume of oxygen.

### Association Between Adipose Tissue Variables and RMR

Supraclavicular PDFF was positively associated with RMR, if data was unadjusted (ß = 11.92 kcal/d, p = 0.026, 95% Cl 1.44 - 22.39 kcal/d), however significance disappeared after adjustment in model 2 and model 3 (model 2: p = 0.346; model 3: p = 0.928) (**Table 2**). No evidence for a statistically significant association between gluteal PDFF and RMR was observed in the unadjusted model (p = 0.389, **Table 2**), while a significantly negative correlation was found in the full adjusted model 3 (ß = -11.60 kcal/d, p = 0.006, 95% Cl -19.75 - -3.45 kcal/d) (**Table 2**). A significantly negative association between delta PDFF and RMR was found in all three models (model 1: ß = -19.41 kcal/d, p = 9.15E-4, 95% Cl -30.65 - -8.16 kcal/d; model 2: ß = -8.17 kcal/d, p = 0.027, 95% CI -15.39 - -0.95 kcal/d; model 3: ß = -8.25 kcal/d, p = 0.023, 95% CI -15.35 - -1.15 kcal/d) (**Table 2**).

**Table 2:** Association between parameters of adipose tissue and RMR.

Parameter	Model 1		Model 2		Model 3	
	beta (95% CI)	p	beta (95% CI)	p	beta (95% CI)	p
PDFF sup. (%) (n = 92)	11.92 (1.44; 22.39)	*0.026*	3.33 (-3.66; 10.33)	0.346	-0.38 (-8.63; 7.87)	0.928
PDFF glut. (%) (n = 92)	-5.70 (-18.79; 7.38)	0.389	-4.12 (-11.22; 2.98)	0.252	-11.60 (-19.75; -3.45)	*0.006*
Delta PDFF (%) (n=92)	-19.41 (-30.65; -8.16)	*9.15E-4*	-8.17 (-15.39; -0.95)	*0.027*	-8.25 (-15.35; -1.15)	*0.023*
VAT (ml) (n = 89)	7.23E-2 (3.81E-2; 0.11)	*6.52E-5*	0.03 (8.58E-3; 0.06)	*0.009*	–	–
SAT (ml) (n = 89)	7.98E-3 (-3.58E-3; 1.95E-2)	0.174	7.10E-3 (3.54E-6; 0.01)	0.050	–	–
FM (kg) (n = 92)	1.87 (-2.53; 6.26)	0.401	3.01 (-0.12; 6.13)	0.059	–	–
FFM (kg) (n = 92)	17.22 (14.95; 19.49)	*<2.0E-16*	19.67 (15.09; 24.25)	*3.95E-13*	17.11 (11.87; 22.34)	*5.24E-9*

Unstandardized beta estimates and 95% confidence intervals for associations between RMR and MRI data, FFM and FM are shown. Model 1 is unadjusted; model 2 is adjusted for age, sex, weight change, FFM (when appropriate); model 3 is adjusted for model 2 plus FM; delta PDFF, difference between PDFF glut. and PDFF sup.; FFM, fat-free mass; FM, fat mass; glut., gluteal; MRI, magnetic resonance imaging; n, number of participants; p, p-value; PDFF, protein-density fat-fraction; RMR, resting metabolic rate; SAT, subcutaneous adipose tissue; sup., supraclavicular; VAT, visceral adipose tissue. Italic p values show a significant association between RMR and parameters of adipose tissue (p ≤ 0.05).

Evidence for a significant association between VAT and RMR was observed in the statistical models 1 and 2 (model 1: ß = 7.23E-2 kcal/d, p = 6.52E-5, 95% CI 3.81E-2 - 0.11 kcal/d; model 2: ß = 0.03 kcal/d, p = 0.009, 95% CI 8.58E-3 - 0.06 kcal/d) (**Table 2**). No significant association was found between SAT and RMR in models 1 and 2 (p ≥ 0.05) (**Table 2**). Furthermore, a significant association was seen between gluteal and supraclavicular PDFF and VAT after adjustment for age, sex, FFM, and weight change (p = 1.44E-5, p = 2.22E-6) (data not shown). Similar results were found for gluteal PDFF, supraclavicular PDFF, delta PDFF, and SAT (data not shown).

### Sub-Analysis in Participants With Normal Weight

To exclude effects of overweight and obesity on the relationship between PDFF and RMR, a sub-group analysis of participants with normal weight (BMI 18.5 – 24.9 kg/m², n = 56) was performed. Evidence for a statistically significant negative association between gluteal PDFF and RMR was obtained in the unadjusted model 1 (ß = -16.30 kcal/d, p = 0.019, 95% CI -29.83 – -2.78 kcal/d) as well as in the full adjusted model 3 (ß = -14.05 kcal/d, p = 0.005, 95% CI -23.67 – -4.43 kcal/d) (**Table 3**). Furthermore, in the unadjusted model 1, an association between FM and RMR was found in people with normal weight (ß = -11.24 kcal/d, p = 0.016, 95% CI -20.32 – -2.15 kcal/d), while no association was found in model 2 (p = 0.221) (**Table 3**).

**Table 3:** Association between parameters of adipose tissue and RMR in persons with BMI 19.5 – 24.9 kg/m^2^.

Outcome	Model 1		Model 2		Model 3	
	beta (95% CI)	p	beta (95% CI)	p	beta (95% CI)	p
PDFF sup. (%) (n=56)	-6.86 (-22.24; 8.52)	0.375	-0.79 (-10.95; 9.37)	0.877	-3.17 (-13.84; 7.50)	0.553
PDFF glut. (%) (n = 56)	-16.30 (-29.83; -2.78)	0.019	-8.24 (-17.17; 0.68)	0.070	-14.05 (-23.67; -4.43)	*0.005*
Delta PDFF (%) (n = 56)	-8.45 (-21.01; 4.11)	0.183	-6.77 (-15.28; 1.73)	0.116	-7.86 (-16.36; 0.65)	0.069
VAT (ml) (n = 53)	0.06 (-0.06; 0.18)	0.302	-0.01 (-0.01; 0.07)	0.749	–	–
SAT (ml) (n = 53)	-7.65E-3 (-0.03; 0.02)	0.534	-1.61E-4 (-0.02; 0.02)	0.983	–	–
FM (kg) (n = 56)	-11.24 (-20.32; -2.15)	*0.016*	4.94 (-3.07; 12.95)	0.221	–	–
FFM (kg) (n = 56)	15.69 (12.29; 19.09)	*9.88E-13*	15.66 (8.52; 22.79)	*5.50E-5*	14.53 (7.19; 21.87)	*2.25E-4*

Unstandardized beta estimates and 95% confidence intervals for associations between RMR and MRI data, FFM and FM are shown. Model 1 is unadjusted; model 2 is adjusted for age, sex, weight change, FFM (when appropriate); model 3 is adjusted for model 2 plus FM; BMI, body mass index; delta PDFF, difference between PDFF glut. and PDFF sup.; FFM, fat-free mass; FM, fat mass; glut., gluteal; MRI, magnetic resonance imaging; n, number of participants; p, p-value; PDFF, protein-density fat-fraction; RMR, resting metabolic rate; SAT, subcutaneous adipose tissue; sup., supraclavicular; VAT, visceral adipose tissue. Italic p values show a significant association between RMR and parameters of adipose tissue (p ≤ 0.05).

### Association Between *FTO* SNP rs1421085 and RMR and Adipose Tissue

No statistically significant differences between the different *FTO* genotypes and the RMR were seen in the additive as well as in the dominant or recessive genetic models (p > 0.05, data not shown), respectively. No evidence for any significant association between the *FTO* SNP rs1421085 and the adipose tissue variables under investigation was obtained, independent of the genetic model (p ≥ 0.05, additive model: **Table 4**; dominant and recessive model: data not shown). Similar results were found when sub-group analysis in persons with normal weight were performed (p ≥ 0.05, data not shown).

**Table 4:** Per risk allele change of the FTO rs1421085 genotype and parameters of adipose tissue.

Parameter	Model 1		Model 2		Model 3	
	beta (95% CI)	p	beta (95% CI)	p	beta (95% CI)	p
PDFF sup. (%) (n=90)	0.02 (-0.01; 0.05)	0.246	0.01 (-0.03; 0.05)	0.604	6.31E-3 (-0.04; 0.05)	0.792
PDFF glut. (%) (n=90)	0.02 (-0.01; 0.06)	0.217	0.02 (-0.02; 0.06)	0.285	0.02 (-0.03; 0.07)	0.356
Delta PDFF (%) (n=90)	-2.81E-3 (-0.04; 0.03)	0.878	0.01 (-0.03; 0.05)	0.556	0.01 (-0.03; 0.06)	0.561
VAT (ml) (n=87)	2.09E-5 (-9.11E-5; 1.33E-4)	0.711	-2.76E-5 (-1.67E-4; 1.12E-4)	0.696	–	–
SAT (ml) (n=87)	1.06E-5 (-2.43E-5; 4.56E-5)	0.546	-2.80E-6 (-4.30E-5; 3.74E-5)	0.890	–	–
FM (kg) (n=90)	0.01 (-5.41E-3; 0.02)	0.252	4.75E-3 (-0.01; 0.02)	0.592	–	–
FFM (kg) (n=90)	4.13E-3 (-0.01; 0.02)	0.514	0.02 (-0.01; 0.04)	0.179	0.01 (-0.02; 0.04)	0.381

Unstandardized beta estimates and 95% confidence intervals for associations between FTO SNP rs1421085 and MRI data, FFM and FM are shown. Model 1 is unadjusted; model 2 is adjusted for age, sex, weight change, FFM (when appropriate); model 3 is adjusted for model 2 plus FM; delta PDFF, difference between PDFF glut. and PDFF sup.; FFM, fat-free mass; FM, fat mass; FTO, fat mass and obesity associated; glut., gluteal; MRI, magnetic resonance imaging; n, number of participants; p, p-value; PDFF, protein-density fat-fraction; SAT, subcutaneous adipose tissue; SNP, single nucleotide polymorphism; sup., supraclavicular; VAT, visceral adipose tissue.

## Discussion

To investigate the effect of BAT on human energy expenditure, we studied the association between supraclavicular PDFF and RMR in a sound group of healthy adults. The main finding was that there was no evidence for an association between mean supraclavicular PDFF and RMR, indicating – if supraclavicular PDFF is considered as a surrogate marker of BAT – that there might be no effect of non-activated BAT on RMR.

### Associations Between MRT-Derived Fat Fractions and RMR

Statistically significant evidence was seen for a negative association between gluteal PDFF and RMR after adjusting for FFM and FM. These findings were similar for males and females (data not shown). Moreover, the negative association between delta PDFF and RMR remained statistically significant after adjusting for age, sex, weight change, FFM and FM. However, the negative correlation disappeared after exclusion of persons with overweight and obesity. These results were in contrast to our hypothesis as we assumed (I) the lower the supraclavicular PDFF, the higher the content of BAT and beige fat cells (II) resulting in a higher delta PDFF and in a higher RMR. The associations may indicate that the adipose tissue composition of the supraclavicular region might not be relevant for energy expenditure, at least under thermoneutrality. Inverse correlations between outdoor temperature and BAT activity were found in different studies, in which BAT was detected *via* PET-CT (29, 30). Moreover, active BAT induced *via* cold expression could generate up to 15 times as much glucose intake than under thermoneutrality (31) leading to an increased energy expenditure. Therefore, other factors influencing RMR like FFM and FM might mask a possible association between BAT and RMR under thermoneutrality.

The significant difference between supraclavicular and gluteal PDFF as demonstrated in this study is in line with other studies (13, 14) indicating a difference in tissue composition concerning the proportions of brown/beige adipose tissue and WAT. In fact, the supraclavicular PDFF is known to be confounded by partial voluming effects due to the presence of both white and brown adipocytes in the human supraclavicular fossa (32). In addition, the gluteal PDFF is known to be positively associated with obesity markers (16). Delta PDFF has been thus proposed as a way to correct for the inter-individual variation of WAT PDFF (14). In the present work, participants with normal weight had a significantly lower supraclavicular PDFF and higher delta PDFF compared to people with overweight or obesity (data not shown). This finding is in line with the recently described positive association between adipose tissue PDFF and obesity markers suggesting that with increasing BMI the relative content of BAT and beige adipocytes is decreasing (16). Similar results have been found in other studies (11, 33). A large retro-perspective study based on 18F-fluorodeoxyglucose PET-CT scans of patients with cancer identified 5 070 participants (9.5% of all participants) with detectable BAT (11). In this study, a significantly inverse correlation between active BAT and BMI (11) suggested that functional BAT is decreasing with increasing BMI. This observation in patients with cancer might indicate lower BAT volume and activity in obesity and is in line with our findings and setting (without control of body temperature and without activation of BAT by drugs). Therefore, we analyzed the association between parameters of adipose tissue and RMR in a sub-analysis in persons with normal weight. No significant association could be found except for a negative association between gluteal PDFF and RMR after adjusting for age, sex, FFM, FM, and weight change. As mentioned before, this negative finding suggests that the volume of BAT might not be associated with RMR.

### Associations Between Body Composition and RMR

From previous studies, it is well known, that FFM is the major determinant of RMR (1, 3, 26, 34), which was also observed in our study. We also found a positive association between VAT and RMR, while SAT and FM were not associated with RMR, both after adjusting for FFM, age, sex and weight change. A recently published MRI study described a significantly higher RMR in persons in whom VAT was pronounced (“visceral obesity”) in comparison to persons with a preferential fat deposition in SAT (35). These associations support the present findings which might be explained by an enhanced metabolic activity of VAT compared to SAT (36). In contrast, Genske et al. identified an association between SAT and RMR independent of VAT volume and adipokines (37). Other studies identified an association between FM and RMR (7, 27, 38) which could not be replicated in the present cohort. However, VAT and SAT, respectively, were significantly associated with FM after the adjustment for age, sex, and FFM (data not shown). These results might be explained that MRI data allows the delineation of specific fat depots, whereas BIA gives an overall FM.

### Associations Between the *FTO* SNP rs1421085 and Fat Compartments

Based on the findings of Claussnitzer et al. (22), the investigation of the relationship between PDFFs or delta PDFF and the C risk allele of the *FTO* SNP rs1421085 was of interest. No association was detected between the rs1421085 obesity C risk allele and PDFFs. However, this negative finding should be dealt with caution as the sample size was limited and, therefore, the number of homozygous C risk allele carriers was rather small. Another explanation might be that the effect of the *FTO* risk genotype may only become apparent when BAT is metabolically active, e.g. after activation by cold exposure. As MRI-derived PDFFs do not reflect current BAT activity, an association between the *FTO* genotype and BAT activity is not excluded and may require further studies.

### Strengths and Limitations

The present analysis investigated the relationship between PDFF as a proxy of BAT and RMR in humans. However, PDFF is not specific for the presence and the proportions of brown or beige adipose cells in a defined adipose tissue depot. A low PDFF in the supraclavicular adipose tissue may suggest a large proportion of less lipid-loaded brown fat cells, but suffers from partial volume effects and cannot reflect the real volume of brown adipocytes in the human supraclavicular fossa comprising a heterogeneous mixture of brown and white adipocytes (16). In addition, PDFF does not provide functional characteristics of the region, which may be critical for local energy expenditure. Despite we have a large sample size compared to other studies including MRI data, the sample size of 92 participants is rather small for the genetic analysis and the generalizability of the results. A strength of the current analysis is the comprehensive phenotyping of participants including body composition analysis, obtained under standardized conditions. In this context, it is noteworthy that the relevance of adjustment for FFM in analyses of fat compartments like VAT or SAT or even for PDFF maps is indistinct. Other studies on the association between BAT volumes and RMR performed no adjustment for FFM (19, 39). It has to be mentioned that BIA differentiates between FM and FFM according to a two-compartment model (40). This approach does not consider the discussion about functional body composition analyses. BAT might be a mixture of FM and FFM and it is rather unclear how to differentiate BAT volumes (41).

## Conclusion

In conclusion, our analysis is the first to investigate the associations between PDFF, as a surrogate marker for BAT and beige fat cells, and RMR and the *FTO* SNP rs1421085. Our results did not provide evidence for a significant association between supraclavicular PDFF and RMR. In addition, our analysis did not reveal an association between supraclavicular PDFF and the *FTO* genotype. Future studies on the association between BAT and RMR should consider the activation of BAT.

## Data Availability Statement

The raw data supporting the conclusions of this article will be made available by the authors, without undue reservation.

## Ethics Statement

The studies involving human participants were reviewed and approved by Ethical committee of the Technical University of Munich. The patients/participants provided their written informed consent to participate in this study.

## Author Contributions

HH, DK, and CHo contributed to conception and design of the study. TD and DJ organized the database. TD, WM, CHe, and DJ performed data collection and analysis. TD and SB performed the statistical analysis. TD, SB, and CHo wrote the manuscript. All authors contributed to the article and approved the submitted version.

## Funding

Else Kroener-Fresenius-Foundation, Bad Homburg, Germany, and the Helmholtz cross-programme topic “Metabolic Dysfunction”.

## Conflict of Interest

DK receives grant support from Philips Healthcare.

The remaining authors declare that the research was conducted in the absence of any commercial or financial relationships that could be construed as a potential conflict of interest.

## Publisher’s Note

All claims expressed in this article are solely those of the authors and do not necessarily represent those of their affiliated organizations, or those of the publisher, the editors and the reviewers. Any product that may be evaluated in this article, or claim that may be made by its manufacturer, is not guaranteed or endorsed by the publisher.
